# Development of a Dispersive µSPE Method for the Determination of Pesticide Residues in Water Samples by LC-MS/MS

**DOI:** 10.3390/molecules31111826

**Published:** 2026-05-26

**Authors:** Gabrielle D. Pereira, Igor F. de Souza, Luana Floriano, Osmar D. Prestes, Renato Zanella

**Affiliations:** Chemistry Department, Federal University of Santa Maria (UFSM), Santa Maria 97105-900, RS, Brazil; diaspereiragabrielle@gmail.com (G.D.P.); igorfdesouza12@gmail.com (I.F.d.S.); luanaflorianoqmc@gmail.com (L.F.); osmar.prestes@ufsm.br (O.D.P.)

**Keywords:** pesticides, water, DµSPE, miniaturization, LC-MS/MS, green analytical chemistry

## Abstract

The increasing occurrence of pesticides in aquatic environments has raised concern due to their potential impact on human health and ecosystems. In this context, the development of sensitive, reliable, and environmentally sustainable analytical methods is essential for monitoring these contaminants. Therefore, the aim of this study was to develop and validate a miniaturized dispersive solid-phase extraction (DµSPE) method for the determination of current-use multiclass pesticides in water samples using liquid chromatography coupled with tandem mass spectrometry (LC-MS/MS). Initially, a simple and rapid sample preparation procedure was developed, in which different experimental variables were evaluated to obtain suitable extraction efficiency. The validated method has a quantification limit of 0.01 µg L^−1^ and was applied to the determination of pesticides in surface water from different regions in Rio Grande do Sul State, Brazil. In addition, the environmental sustainability of the method was evaluated using the AGREEprep tool, allowing a quantitative and visual assessment of its compliance with the principles of Green Analytical Chemistry. The results demonstrated that the proposed method provides adequate analytical performance for the determination of 28 compounds in water matrices while offering a simple sample preparation procedure with reduced solvent consumption and waste generation.

## 1. Introduction

Water is a natural resource essential to sustaining life and maintaining the balance of ecosystems and has been recognized since ancient times as a fundamental element for the existence of living organisms. Although approximately 71% of the Earth’s surface is covered by water, only an extremely small fraction is available for human use. More recent data indicate that millions of people still lack access to safe drinking water and adequate sanitation, highlighting that the water crisis is already a global reality [1]. Among the sectors with the highest water demand, agriculture stands out, accounting for approximately 70% of global water consumption [2]. Although irrigation has led to significant gains in food production, it has also contributed to environmental degradation and the contamination of soil and water bodies, predominantly with pesticides.

The growing demand for water resources observed over the past decades is directly linked to population growth, the intensification of productive activities, and urban expansion. In Brazil, CONAMA Resolution No. 357/2005 established quality standards for surface waters [3]. For drinking water, GM/MS Ordinance No. 888/2021 established maximum permissible limits for various chemical parameters, including pesticides and their metabolites [4]. In addition, the state of Rio Grande do Sul (Brazil) established the SES/RS Ordinance No. 320/2014, which expands the scope of regulated pesticides in water intended for human consumption [5]. Under Brazilian legislation, except for organochlorine pesticides, which have very low limits, the maximum permitted levels for other pesticides are above 0.1 µg L^−1^. In the European Union (EU), the Water Framework Directive [6] established parameters for sustainable water management, followed by successive directives and decisions that included several substances subject to monitoring. Additionally, the Drinking Water Directive [7] set parameters for many pollutants, with a limit of 0.1 µg L^−1^ for each pesticide. Given this regulatory framework and the widespread use of pesticides in agriculture, the monitoring and determination of residues of these compounds in water samples are essential for assessing environmental impacts and informing more effective management and control measures [8,9,10].

The presence of pesticide residues in the environment occurs through various pathways, including drift during application, surface runoff, leaching into groundwater, and volatilization processes influenced by both the physicochemical properties of the compounds and edaphoclimatic factors. Once introduced into aquatic ecosystems, these contaminants can affect non-target organisms, promote ecological imbalances, reduce biodiversity, and trigger processes of bioaccumulation and biomagnification along food chains [11,12]. Additionally, human exposure can occur through the consumption of contaminated water and food, with toxicological effects depending on the substance, dose, and duration of exposure, potentially resulting in acute or chronic poisoning [13]. Given these risks, water quality monitoring has become a priority in environmental and health public policies.

Although often detected at concentrations in the order of µg L^−1^ or lower, the environmental persistence and toxic potential of pesticides require highly sensitive, selective, and robust methods [14]. Chromatographic techniques coupled with mass spectrometry have stood out in the determination of environmental pollutants due to their high sensitivity and selectivity [15]. Liquid chromatography coupled with tandem mass spectrometry (LC-MS/MS) represents one of the most advanced approaches in this field, combining high chromatographic resolution, analytical speed, and excellent performance in the quantification of organic compounds [16]. The use of triple-quadrupole analyzers and electrospray ionization (ESI) sources enables the selection of precursor ions and the generation of product ions, significantly increasing the specificity and reliability of the analyses. However, the success of these determinations depends heavily on the sample preparation step, which is responsible for concentrating the analytes and removing matrix interferents. Classic techniques, such as liquid–liquid extraction, have given rise to more selective and efficient approaches, including solid-phase extraction (SPE) and miniaturized methods. SPE is widely used in the analysis of pesticide residues in water and is recommended by regulatory agencies due to its ability to concentrate analytes and its robustness [17,18]. SPE is based on the selective retention of analytes on specific sorbents contained in cartridges, followed by elution with small volumes of solvent [19]. Despite its effectiveness, SPE involves considerable material costs that can be reduced by miniaturizing the sample preparation step. Micro solid-phase extraction (µSPE) is a miniaturized technique that has been successfully used, particularly during the cleanup stage of extracts from the QuEChERS method for the determination of pesticides in food samples [20,21,22,23,24] and as a preconcentration step to evaluate cyclophosphamide and iphosphamide in urine [25]. Dugheri et al. [26] presented a review of µSPE and devices applied in sample preparation. A variation in µSPE is the dispersive µSPE (DµSPE) technique, which has been used to determine various compounds in different matrices due to its simplicity of operation, lower material consumption, and minimal waste generation. DµSPE has been employed in the determination of various compounds in different matrices, such as: polycyclic aromatic hydrocarbons in water samples [27,28,29], N-nitrosamines in swimming pool water [30], synthetic polycyclic musks in water [31], benzodiazepines from biological fluids [32], phenolic compounds in chrysanthemum [33], and benzophenone-type ultraviolet absorbers in aqueous samples [34]. More recently, this technique has also been applied for the determination of per- and polyfluoroalkyl substances in environmental river water [35].

There are some DµSPE applications for the preconcentration of pesticides from water samples, most of which focus on only one class of pesticide and a few compounds. Chen et al. [36] applied a polymer cation exchange (PCX) for the determination of triazine herbicides in drinking water by DµSPE. The method used 35 mg of PCX and achieved limits of detection (LODs) ranging from 0.2 to 30.0 ng L^−1^ for the 30 triazines. Galán-Cano et al. [37] used ionic liquid-modified silica for the determination of four organophosphate pesticides in water by liquid chromatography with a diode array detector. The LODs ranged from 0.3 to 0.6 μg L^−1^. Akbarzade et al. [38] applied reduced graphene oxide quantum dots as a magnetic sorbent for the extraction of five organophosphorus pesticides in water, followed by determination using gas chromatography-mass spectrometry (GC-MS). The LODs were less than 0.07 µg L^−1^. Kermani et al. [39] used porous magnetized carbon sheet nanocomposites for the determination of three organophosphorus pesticides by GC-MS with LODs ranging from 0.46 to 1.00 μg L^−1^. Jiang et al. [40] described a method based on DµSPE for the analysis of eight organochlorine pesticides in aqueous samples by GC-MS. The LODs ranged from 0.0121 to 0.0468 μg L^−1^.

Alongside advances in analytical techniques, there is growing concern about the environmental impacts associated with laboratory practices. In this context, Green Chemistry and, more specifically, Green Analytical Chemistry (GAC) propose the development of methods that reduce the consumption of toxic solvents, waste generation, and energy use, without compromising the quality of results [41]. The twelve principles of GAC were proposed by Anastas and Warner [42], establishing a conceptual foundation for the development of sustainable practices. Metrical tools, such as AGREEprep, allow for the objective assessment of the sustainability of sample preparation procedures [43]. The application of these metrics makes it possible to identify critical points and guide the optimization of methods from an environmental perspective. There is a clear need for analytical methods capable of determining pesticide residues in environmental water samples with high sensitivity, selectivity, and reliability, combined with more sustainable sample preparation practices.

In this context, the present study aimed to develop and validate a multiresidue method for the determination of current-use pesticides in water samples using a miniaturized sample preparation technique based on miniaturized dispersive solid-phase extraction (DµSPE) and LC-MS/MS, as well as its application to water samples, with the aim of contributing to water quality monitoring and the assessment of environmental impacts associated with the use of these compounds. The pesticides selected for this study have been widely used in agriculture and include herbicides, fungicides, insecticides, acaricides, and nematicides.

## 2. Results

### 2.1. LC-MS/MS Established Conditions

The LC-MS/MS analytical method used in this study was established based on the optimization of analytical conditions, with the aim of obtaining the highest instrumental response for the compounds evaluated. Chromatographic separation using a biphenyl column showed good efficiency in separating the analytes, which was performed using gradient elution, with a total analysis time of 8.5 min. Table S1 presents the LC-MS/MS acquisition parameters, including the respective retention times and monitoring transitions. Two precursor–product ion transitions were monitored for each analyte; the one with the highest intensity was selected for quantification, while the second most intense was used for analyte confirmation. The final extract did not need to be diluted, which helped achieve lower detection limits. Figure S1 shows the Total Ion Chromatogram (TIC) obtained by LC-MS/MS of the lower point (0.1 µg L^−1^) of the extracted curve, that correspond to 0.01 µg L^−1^ in the water sample.

### 2.2. Evaluation of the Sample Preparation Method

The main parameters influencing the efficiency of the sample preparation step were evaluated. Factors such as extraction conditions, sample volume, and the concentration step can directly impact the recovery of analytes. Thus, the most relevant variables associated with sample preparation were investigated to verify their influence on extraction efficiency and the analytical response obtained. The evaluation of these parameters allowed for the identification of the most appropriate conditions for sample preparation, contributing to the reliability of the analyses performed.

**Figure 1:**
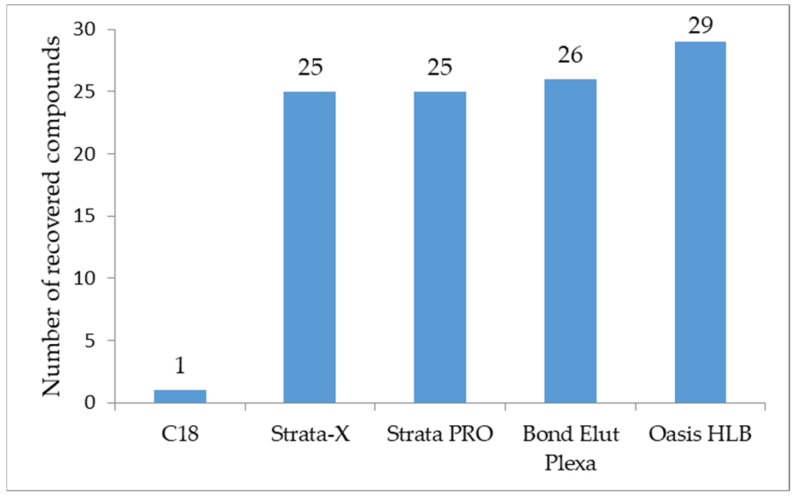
Comparison of the performance of the different sorbents evaluated in the dispersive extraction step, expressed as the number of compounds recovered for each tested material.

The performance of different sorbents in dispersive extraction was evaluated to identify the material that provided the highest efficiency in analyte retention. The polymeric sorbents Oasis HLB, Strata-X PRO, Bond Elut Plexa, and Strata-X were tested, in addition to the C18 sorbent based on chemically modified silica, all of which exhibit distinct physicochemical characteristics and, consequently, different mechanisms of interaction with organic compounds. C18 is hydrophobic and, during extraction, this sorbent tended to float on the surface of the water, resulting in poor analyte extraction. In general, polymeric sorbents such as Oasis HLB and Strata-X are widely used in the extraction of organic compounds due to their high versatility, as they combine hydrophobic and hydrophilic interactions, favoring the retention of compounds with different polarities. The efficiency of each evaluated material can be seen in Table S2 and Figure 1, which shows the number of compounds recovered for each of the sorbents used.

It can be observed that the Oasis HLB sorbent yielded a significantly higher number of recovered compounds compared to the other sorbents evaluated. This result may be related to the characteristics of the sorbent, which has a polymeric matrix with a hydrophilic-lipophilic balance, favoring multiple types of interaction with the analytes and, consequently, more efficient retention of compounds with different polarities. Given this superior performance, Oasis HLB was selected for the subsequent stages of method optimization and application. Oasis HLB yielded adequate results for pesticides in water using rotating disk sorptive extraction (RDSE) [44] and cartridge SPE [45,46] with LC-MS/MS detection.

The evaluation of sorbent mass is a critical factor in the application of dispersive techniques, since the amount of the extractant phase directly influences the retention capacity of the analytes and, consequently, the extraction efficiency. Reduced sorbent masses (2, 6, and 10 mg) exhibited unsatisfactory performance, characterized by low precision among replicates and limited recovery of compounds. This behavior can be attributed both to the lower available sorption capacity and to the operational difficulties associated with handling very small masses. A low sorbent mass can result in lower retention of the compounds and greater variability in the observed recoveries. Increasing the sorbent mass resulted in a significant improvement in the method’s performance. The use of 20 mg allowed for the recovery of 23 compounds, while 30 mg enabled the recovery of 28 compounds, indicating greater efficiency of the extraction process. Although the herbicide metsulfuron-methyl showed acceptable recovery using Oasis HLB in the sorbent selection stage, in the other tests, recoveries were below 60%. This herbicide showed adequate recoveries in the study described by Quesada-Molina et al. [47], but for much higher concentration levels and with a 60 mg cartridge. The improvement in analyte recovery with the higher amount of sorbent can be explained by the greater availability of active interaction sites and by the behavior of the material during centrifugation. Under these conditions, better sorbent sedimentation and less material loss during handling were observed, factors that contribute to greater precision of the procedure.

**Figure 2:**
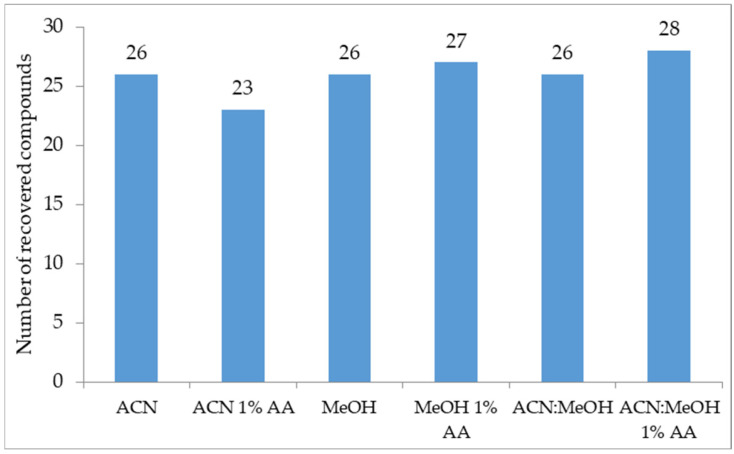
Relationship between the desorption solvents evaluated and the compounds that exhibited recoveries between 60 and 130% at a concentration of 0.1 µg L^−1^.

The tests to evaluate the desorption solvent were performed using a blank sample spiked at 0.1 µg L^−1^. Figure 2 and Table S3 shows the number of compounds recovered as a function of the desorption solvent composition, highlighting the direct influence of this parameter on the efficiency of the extraction process. In general, it was observed that pure solvents performed worse when compared to solvent mixtures. This behavior may be related to the structural diversity of the analytes evaluated, which exhibit different polarities and chemical characteristics, hindering efficient elution using a single solvent. Among the conditions tested, mixtures of acetonitrile and methanol performed best, especially when acidified with acetic acid. Acidification of the medium may promote desorption by reducing electrostatic interactions and facilitating the release of retained compounds. This behavior is consistent with previously reported results in the literature [48], which indicate that acidified ACN:MeOH mixtures exhibit greater efficiency in the elution of organic compounds. Among the conditions evaluated, the ACN:MeOH mixture acidified with 1% (*v*/*v*) acetic acid showed the best performance in the recovery of the compounds studied and was selected for conducting the subsequent experiments.

Given the importance of pH adjustment for extraction efficiency, three distinct conditions were evaluated: acidic medium (pH ≈ 2.5), sample without pH adjustment (pH ≈ 7), and basic medium (pH ≈ 9). Sample acidification was performed using 1:1 (*v*/*v*) H_3_PO_4_, while 5% (*v*/*v*) NH_4_OH was used for the basic condition. All assays were conducted with spiked blank samples at a concentration of 0.1 µg L^−1^. At pH 2.5, 28 compounds were recovered; at neutral pH, 27; and at pH 9.0, only 24 compounds. Based on the results obtained, it was observed that the acidic pH condition favored interaction with the sorbent, yielding the highest recoveries.

**Figure 3:**
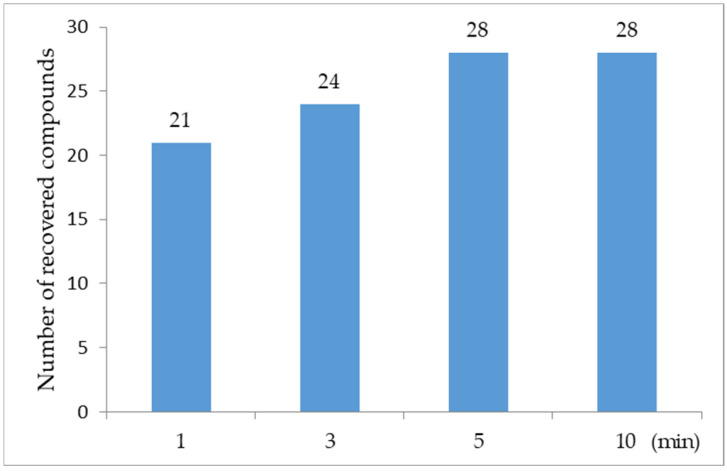
Compounds that exhibited average recoveries between 60 and 130% and RSD ≤ 20% as a function of the different sonication times evaluated.

The evaluation of the efficiency of the analyte extraction step from the sorbent using vortex mixing (2800 rpm) and ultrasound (40 kHz frequency and 135 W power) indicated that ultrasound-assisted extraction performed better (28 compounds) compared to vortex mixing (26 compounds). The result suggests that the energy provided by ultrasound promoted greater mass transfer in the system, favoring the desorption of compounds retained in the solid phase. Thus, ultrasound was selected for the subsequent stages of desorption process optimization. The evaluation of sonication time (1, 3, 5, and 10 min) indicated that 5 and 10 min yielded similar results (Figure 3), suggesting that 5 min was sufficient.

### 2.3. Validation of the Method

Table 1 presents the coefficients of determination (r^2^) for the extracted curve obtained in the range of 0.05 to 1.0 µg L^−1^. Adequate analytical responses were obtained with r^2^ > 0.99 for all compounds evaluated. Results of accuracy and precision, evaluated through the recovery and RSD of blank samples spiked at 0.01, 0.02, and 0.1 μg L^−1^, are presented in Table 1. The accuracy and precision, in terms of repeatability and intermediate precision, yielded acceptable results for all levels in accordance with SANTE [49] criteria (recoveries from 70 to 120% and RSD ≤ 20%), with exception of the herbicide metsulfuron-methyl that did not yield adequate recovery results and could not be validated. The high polarity of this herbicide (log Kow −1.87) explains the low recoveries. Considering that the method limit of quantification (LOQ) corresponds to the lowest spiking level that present satisfactory performance, with recoveries from 70 to 120% and RSD ≤ 20%, all validated compounds presented a LOQ of 0.01 µg L^−1^. The LOD, obtained by considering one-third of the LOQ value, is 0.003 µg L^−1^. These method limits were very satisfactory in relation to the limits established in Brazilian and European regulations and, in general, are lower than the values obtained with similar published methods [36,37,38,39,40]. The use of the DµSPE technique for sample preparation requires a smaller amount of sorbent and elution solvents than similar methods, showing good performance in terms of selectivity, sensitivity, precision and accuracy for 28 pesticide residues from different classes. The proposed method does not require the manifold system and vacuum pump necessary in SPE methods. Thus, the developed method has great potential for routine laboratory use and can be employed to monitor these pesticides in water samples.

### 2.4. Environmental Sustainability Using the AGREEprep Tool

**Figure 4:**
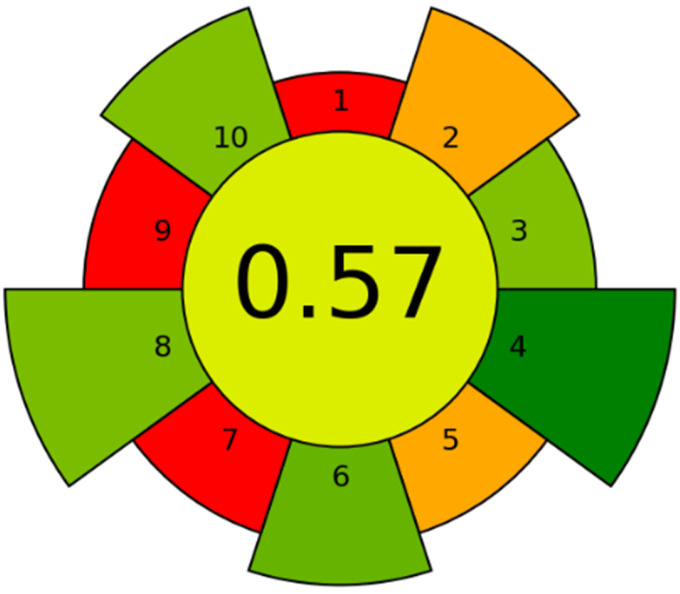
Assessment of the sustainability criteria of the analytical method using the 10 criteria of the AGREEprep tool.

The greenness metric for sample preparation (AGREEprep) was assessed using the online calculator established by Wojnowski et al. [43]. The higher the AGREEprep score, the greener the method. The proposed method is classified as ex situ, since samples are collected and transported to the laboratory, resulting in the lowest score assigned to the first criterion. Regarding the second criterion, which concerns the use of safe solvents, the method uses 1 mL of toxic solvents, resulting in a moderate penalty. In the third criterion, the method allows for the reuse of Falcon tubes and uses a small amount of sorbent compared to the traditional method and without disposable cartridges, representing an advance in terms of reducing consumables. In the fourth criterion, related to minimizing waste generation, the metric assigns the maximum score to methods that generate less than 0.1 g or mL of waste, which is the case for the proposed method. In the fifth criterion, which considers the miniaturization of the method, the use of only 10 mL of sample characterizes it as miniaturized. In the proposed method, it is possible to prepare 30 samples per hour, which represents a satisfactory processing rate under the sixth criterion. In the seventh criterion, which considers the degree of automation of the procedure, the developed method is entirely manual, resulting in a low score for this criterion. The eighth criterion evaluates the method’s energy demand. For this analysis, the total electrical energy consumption of the equipment used was estimated based on the nominal power and the effective operating time, and subsequently divided by the number of samples processed, in order to express energy consumption in watt-hours per sample (Wh/sample). The sonication step was performed in an ultrasonic bath with an approximate nominal power consumption of 60 W for 5 min for the simultaneous processing of 30 samples. Thus, the energy consumed was 5.0 Wh, resulting in a consumption of approximately 0.17 Wh per sample. The centrifugation step was performed in a centrifuge with a nominal power of 1500 W. The experimental protocol consisted of two centrifugation cycles, performed at 6000 rpm, each lasting 8 min, totaling 16 min of operation for the same set of 16 samples. The total operating time was converted to hours, resulting in a total energy consumption of approximately 400 Wh. Considering the processing of 16 samples, the energy consumption per sample during centrifugation was 25 Wh per sample. The ninth criterion refers to the instrumental techniques and measurements employed, which in this case requires the use of advanced analytical instrumentation, contributing to a lower score. Finally, the tenth criterion assesses the risk to the operator. In the developed method, the main risk identified is associated exclusively with the use of toxic organic solvents. Thus, only one risk factor is considered for the evaluation of this criterion. Figure 4 presents the results of the evaluation of the analytical method using the AGREEprep tool.

The overall score of 0.57 obtained by AGREEprep indicates that the developed method exhibits intermediate environmental performance, reflecting a balance between favorable sustainability aspects and limitations inherent to the adopted analytical procedure. Among the factors that contributed positively to the score, the high miniaturization of the method stands out, as it adheres to the principles of Green Analytical Chemistry, significantly reducing reagent consumption and waste generation. Another positive aspect relates to the method’s throughput, which allows for the simultaneous preparation of a large number of samples per hour, reducing operator exposure time and contributing to greater operational efficiency. Occupational risk is also considered limited, since the main risk factor is associated only with the use of organic solvents in small volumes. Thus, the value of 0.57 reflects a method that incorporates important principles of sustainability, such as miniaturization, solvent reduction, and low waste generation, but which still has limitations associated with ex situ sampling, the manual nature of the procedure, energy demand, and the use of sophisticated analytical instrumentation. These results indicate that the method is environmentally acceptable, but with potential for future improvements, especially through the automation of steps and the reduction in energy consumption.

### 2.5. Application to Water Samples

The results obtained by applying the proposed method to 20 river water samples from various regions of the state of Rio Grande do Sul, Brazil, are presented in Table 2. Compounds with results below the LOD are indicated as not detected (n.d.), and values between the LOD and the LOQ of the method are reported as <LOQ. The method allowed for accurate determination at very low concentration levels, even when applied to river water samples that generally contain many organic compounds that could hinder the detection of pesticides. The herbicide atrazine and the fungicide tebuconazole are present in most of the samples analyzed. Atrazine is widely used in Brazil, being one of the most applied pesticides in crops such as corn due to its effectiveness in weed control, despite its ban in several countries due to environmental and health concerns. Atrazine’s mobility in soil and its environmental persistence facilitate transport via surface runoff and leaching, which can result in its detection in both surface and groundwater near application sites. Tebuconazole is a fungicide from the triazole group, widely used in agriculture. Figure S2 presents the total ion chromatogram of atrazine for sample S20, the LOQ, and the blank sample.

## 3. Materials and Methods

### 3.1. Reagents and Materials

SPE cartridges containing the following sorbents were used: Oasis HLB 60 mg/3 mL (Waters, Milford, MA, USA); Strata-X PRO 200 mg/3 mL and Strata-X 200 mg/3 mL (Phenomenex, Torrance, CA, USA); Sep Pak C18 500 mg/6 mL; and Bond Elut Plexa 500 mg/6 mL (Agilent Technologies, Santa Clara, CA, USA). Solid standards of the compounds under study were obtained from LGC Standards (Wesel, Germany) with purity higher than 98%; the atrazine-d5 surrogate standard and the reserpine internal standard were from Sigma Aldrich (San Luis, CA, USA). HPLC-grade acetonitrile and methanol (J.T. Baker, Ecatepec, Mexico), ultrapure water purified in a Milli-Q Direct 3UV^®^ system with a resistivity of 18.2 MΩ cm^−1^ (Millipore, L’Isle-d’Abeau-Chesnes, France), anhydrous ammonium formate (Sigma Aldrich, San Luis, CA, USA), formic acid p.a. ≥ 98% (Sigma Aldrich, San Luis, CA, USA), and phosphoric acid p.a. 85% and acetic acid p.a. 99% (Merck, Rio de Janeiro, Brazil) were used. Nylon filters with a diameter of 13 mm and a pore size of 0.22 μm (Chromastore, São Paulo, Brazil), as well as 2 mL vials, 2 mL Eppendorf tubes (Eppendorf, Hamburg, Germany), and various standard laboratory glassware were used.

Standard solutions were prepared individually in acetonitrile at a concentration of 1000 mg L^−1^, considering the purity of the solid standards. From these solutions, a working solution in acetonitrile containing all analytes at a concentration of 5 mg L^−1^ was prepared. To evaluate the method’s linearity and construct the extracted calibration curves, solutions in acetonitrile containing all analytes at concentrations of 10, 100, and 1000 µg L^−1^ were prepared. Next, the extracted analytical curves were obtained by spiking water samples with different concentrations of the analyte mixture at 0.005, 0.01, 0.025, 0.05, and 0.1 µg L^−1^, prior to the extraction process. Considering a 10-fold preconcentration, the concentration in the final extract were 0.05, 0.1, 0.25, 0.5 and 1.0 µg L^−1^. The pesticides selected (Table 1) for this study are compounds widely used in agriculture, including herbicides, fungicides, insecticides, acaricides, and nematicides. Deuterated atrazine (atrazine-d5) was used as a surrogate standard to confirm that sample preparation was performed correctly. It was added (10 µL of the 10 µg L^−1^ solution) prior to the extraction step to the spiked blank samples and the samples analyzed, resulting, after a 10-fold preconcentration, in a final concentration in the analyzed extract of 0.1 µg L^−1^. Reserpine was used as an internal standard, with 10 µL of the 50 µg L^−1^ solution added directly to the final extract prior to LC-MS/MS analysis at a concentration of 0.5 µg L^−1^. The PI was used to verify the stability of the instrument’s response, without the intention of incorporating its response directly into the quantification of the analytes.

### 3.2. Instrumentation

For the analyses, a QTRAP LC-MS/MS system (SCIEX, Redwood City, CA, USA) was used, equipped with an LC-40B X3 CL liquid chromatograph with an automatic injection system, a triple quadrupole MS detector with a linear ion trap (model 6500+), an electrospray ionization (ESI) source (ESI) Turbo V Ion Source, Sciex OS data acquisition software version 3.4, and a model N19A26 nitrogen generator (PEAK Scientific, Inchinnan, Scotland). The following were also used: a 2800 rpm AP56 vortex mixer (Phoenix, Araraquara, Brazil), AUW 220D and UX-420H analytical balances (Shimadzu, Kyoto, Japan), automatic micropipettors with variable capacity (Brand, Hamburg, Germany), an ultrasonic bath model USC-1400 with an ultrasonic frequency of 40 kHz, power of 135 W, and capacity of 2.8 L (Unique, Indaiatuba, Brazil), and an NT 825 centrifuge for 15 mL tubes (Novatécnica, São Paulo, Brazil).

### 3.3. LC-MS/MS Analysis

The chromatographic method utilized a Kinetex^®^ Biphenyl column (100 × 2.1 mm i.d., 2.6 μm) from Phenomenex (USA), maintained at 60 °C, employing as the mobile phase (A) ultrapure water with 2% (*v*/*v*) methanol and (B) methanol with 2% (*v*/*v*) ultrapure water, both containing 0.1% (*v*/*v*) formic acid and 5 mmol L^−1^ ammonium formate. The gradient used was: 0 to 0.25 min 5% B; 0.25 to 7.75 min increase in B to 100% B, maintained until 8.50 min. The mobile phase flow rate was 0.350 mL min^−1^ and the injection volume was 5 µL. The detector used was a triple quadrupole MS operating with an electron spray ionization (ESI+) source in positive mode under the following conditions: capillary voltage: 4 kV; desolvation temperature: 500 °C; source temperature: 300 °C; and dwell time: 10 to 200 ms. The quantification and confirmation transitions in selected reaction monitoring (SRM) mode, as well as the retention time, declustering potential (DP), entrance potential (EP), collision energy (CE), and collision cell exit potential (CXP), are presented in Table S1. The conditions were established to obtain adequate responses from the selected compounds.

### 3.4. Evaluation of Sample Preparation

Several parameters were evaluated to determine the optimal sample preparation conditions. The wide variety of analytes, with log Kow values ranging from −1.87 for metsulfuron-methyl and −0.13 for thiamethoxam to 4.50 for trifloxystrobin, makes sorbent selection challenging in this experiment.

#### 3.4.1. Selection of the Sorbent Used for the Concentration of Analytes

The first step consisted of determining the appropriate mass of sorbent for the application of the dispersive SPE technique. Aiming to miniaturize the procedure, preliminary tests were conducted using 10 mL of tap water sample, free of the compounds under study, spiked at 0.1 µg L^−1^ to ensure comparability among the tested materials, and different masses (2, 6, 10, 20, and 30 mg) of the Oasis HLB sorbent, frequently used to concentrate pesticides in water samples [50,51]. The smaller masses (2, 6, and 10 mg) showed poor reproducibility, especially due to the low amount of sorbent and losses during handling. Thus, larger masses (20 and 30 mg) were prioritized. With 20 mg, 23 compounds were recovered, while 30 mg allowed for the recovery of 29 compounds, demonstrating better performance. This improvement is directly related to the greater stability of the sorbent during centrifugation, better sedimentation, and reduced losses, resulting in more consistent and efficient extractions. In the next step, a comparative evaluation of different sorbents (30 mg) was conducted to identify the one that performed best in dispersive extraction. Materials with distinct characteristics were selected, including the polymeric sorbents Oasis HLB, Strata-X, Strata-X PRO and Bond Elut Plexa, as well as C18. As elution solvent, the ACN:MeOH mixture containing 1% (*v*/*v*) acetic acid (AA) was used, according the work of de Oliveira et al. [48]. A comparison of these materials revealed that Oasis HLB and Bond Elut Plexa exhibit similar efficiencies that are significantly superior to those obtained by Strata-X and Strata-X PRO. This evidence supports the selection of polymeric sorbents as preferred candidates in multiresidue extraction methods, especially when seeking broad structural coverage and stability under different matrix conditions [50,51].

During the preparation of the extraction curve, an unexpected behavior was observed related to the addition of the working solution (WS) prepared in acetonitrile (ACN) at 1 mg L^−1^. Initially, volumes of 10, 25, 50, and 100 µL of the WS were tested; however, complete sedimentation of the sorbent after centrifugation occurred only at the 50 and 100 µL volumes. To systematically evaluate this effect, intermediate volumes (50, 75, and 100 µL) were tested, confirming that, with larger volumes of ACN added to the aqueous medium, the sorbent ceases to remain dispersed and rapidly settles at the bottom of the Falcon tube. To ensure uniformity in the sorbent’s behavior across all points of the curve, it was decided to always use 100 µL of the ACN standards at all points, avoiding unwanted variations in the degree of dispersion. To implement this strategy, a pre-curve ten times more concentrated than the final analytical curve was prepared consisting of an intermediate working solution, prepared in ACN with different concentrations of the analytes, from which aliquots of the same volume (100 µL) were withdrawn for sample spiking, ensuring that the sedimentation process occurred reproducibly at all evaluated concentrations. This experimentally observed phenomenon is consistent with the behavior described in the studies reported by Bouazizi and Ayachi [52], who report that water-ACN mixtures exhibit micro-heterogeneity depending on the ACN fraction. The sudden addition of ACN to the aqueous medium can generate zones of lower density around the organic droplet, causing the denser solvent to settle more rapidly in these higher volumes of organic solvent.

#### 3.4.2. Selection of the Elution Solvent

The selection of the elution solvent is one of the most critical factors for extraction efficiency, since its physicochemical properties directly influence the ability to break the interactions between the analytes and the sorbent. According to de Oliveira et al. [48], the ACN:MeOH mixture containing 1% (*v*/*v*) acetic acid (AA) exhibits superior performance in the elution of drugs, and this behavior can be partially attributed to differences in polarity, viscosity, and eluting power between acetonitrile and methanol. Acetonitrile has lower viscosity (≈0.37 mPa·s) compared to methanol (≈0.54 mPa·s), a characteristic that promotes more efficient cavitation bubble formation and reduces flow resistance in the medium, intensifying desorption processes. Furthermore, the greater eluting power of acetonitrile in reverse-phase systems contributes to the displacement of strongly retained analytes, while methanol complements elution through additional interactions, such as hydrogen bonding and dipole interactions, broadening the spectrum of extracted compounds [53]. Given the high structural diversity of the pesticides evaluated, it was expected that a single solvent would not be effective, justifying the investigation of pure solvents and mixtures, with and without acidification using acetic acid (AA). Thus, ACN, MeOH, ACN 1% (*v*/*v*) AA, MeOH 1% (*v*/*v*) AA, ACN:MeOH, and ACN:MeOH 1% (*v*/*v*) AA were tested. For preliminary evaluation purposes, recoveries between 60 and 130% were considered adequate.

#### 3.4.3. Evaluation of the Effect of Vortexing vs. Ultrasound

To improve the efficiency of the analyte extraction step from the sorbent, vortex mixing (2800 rpm) and ultrasound-assisted extraction (bath with a frequency of 40 kHz and power of 135 W) were evaluated for 1, 3, 5, and 10 min. All experiments were performed using blank samples spiked at 0.1 µg L^−1^; 1 mL of a 1% (*v*/*v*) acetic acid mixture of ACN:MeOH was used as the extraction solvent.

### 3.5. Established Sample Preparation Procedure

**Figure 5:**
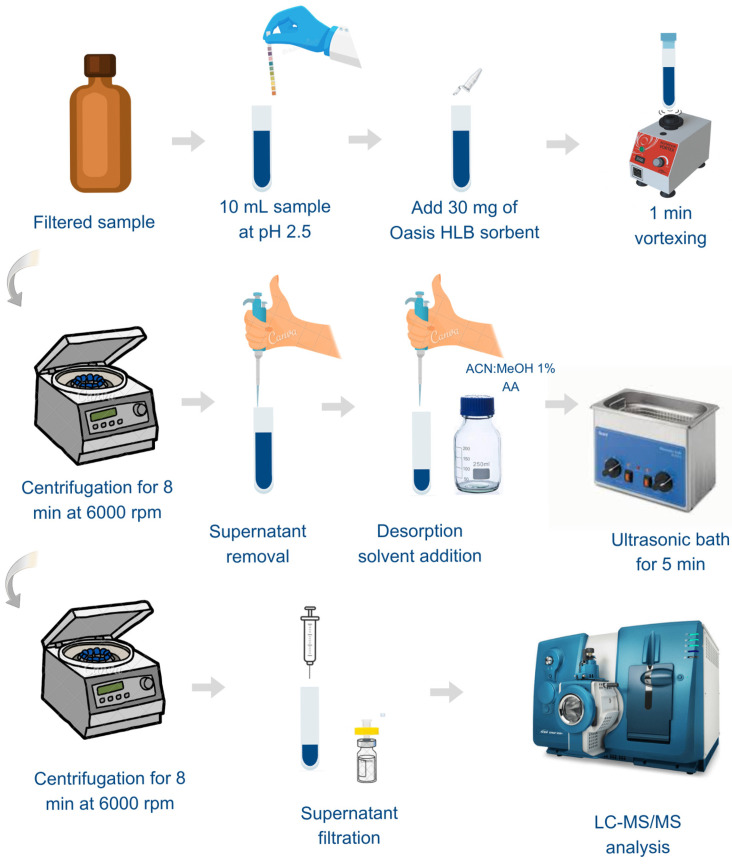
Flowchart of the proposed method.

The established method used 10 mL of sample in a 15 mL Falcon tube, followed by adjustment to pH 2.5 and the addition of 30 mg of Oasis HLB sorbent. The mixture was homogenized by vortexing for 1 min and subsequently centrifuged for 8 min at 6000 rpm to promote sorbent sedimentation. The supernatant was discarded, 1 mL of the 1% (*v*/*v*) ACN:MeOH mixture was added to the sorbent, and the extract was sonicated for 5 min. The tube was centrifuged again; the extract was filtered through a nylon filter (0.22 µm; 13 mm) into a 2 mL vial and analyzed using the LC-MS/MS system. Figure 5 shows the sample preparation procedure established in this study.

### 3.6. Method Validation

Method validation was performed based on the criteria established by the SANTE guideline [49]. The parameters evaluated were precision, linearity, selectivity, accuracy, limit of quantification (LOQ), and limit of detection (LOD). The analytical curve was evaluated using solutions prepared at concentrations of 0.05, 0.1, 0.2, 0.4, 0.6, and 1.0 µg L^−1^. The extracted curves were injected into triplicate. This step aimed to verify the working range and the behavior of the analytical response for each analyte, in addition to evaluating the linearity of the method using the coefficient of determination (r^2^). Table 1 presents the compounds evaluated in this study, with their respective retention times, acquisition windows, monitored transitions for quantification and confirmation, and the main instrumental parameters used in the LC-MS/MS analysis. The use of extracted curves constitutes a fundamental strategy in the validation of methods employing microextraction techniques, as it allows for consideration of the actual behavior of the analytes throughout all stages of the analytical procedure [54]. The need to use extracted curves in microextraction was highlighted by Ghambarian et al. [55], who applied dispersive liquid–liquid microextraction (DLLME) for the determination of nonsteroidal anti-inflammatory drugs in water samples. The authors demonstrated that natural matrix constituents modify the partition efficiency of analytes between phases, directly impacting recoveries. Complementarily, Zhang et al. [56] evaluated the application of SPME in different environmental and biological matrices, observing variations exceeding 15% in the analytical response among the different extracts. Thus, the use of extracted curves is widely recommended in methods employing microextraction.

In this study, accuracy was determined through recovery experiments, using blank samples spiked with the analytes at 0.01, 0.02, and 0.1 µg L^−1^. Precision, expressed in terms of repeatability and intermediate precision, was evaluated by the relative standard deviation (RSD) of the recovery assays, in 7 replicates, on the same day and on different days, respectively. The LOQ was determined by the experimental approach considering the lowest spiking level that showed satisfactory performance considering the SANTE criteria [49], and the LOD was obtained by considering one-third of the LOQ value. As a blank sample, a water sample was used, collected from an area far from agricultural crops. This sample was analyzed to confirm the absence of the analytes under study.

### 3.7. Environmental Sustainability Assessment Using the AGREEprep Tool

The AGREEprep software was applied to the systematic assessment of the toxicity, risks, and environmental performance of analytical methods, considering the different stages of sample preparation [43,57]. The first criterion relates to the sampling stage, the second refers to the use of safe solvents, the third evaluates the use of sustainable, renewable, or reusable materials, the fourth focuses on minimizing waste generation, the fifth relates to the miniaturization of the method and considers sample size, the sixth evaluates the sample processing rate, the seventh considers the degree of automation of the procedure, classifying methods as fully automated, semi-automated, or manual, the eighth evaluates energy demand, the ninth refers to instrumental techniques and measurements, and the tenth evaluates the risk to the operator.

### 3.8. Application of the Validated Method

The developed and validated method was applied to 20 river water samples collected in October 2025 from various regions of the state of Rio Grande do Sul, Brazil. The samples were collected in 50 mL amber vials and kept refrigerated until analysis, which was performed in the same week as collection.

## 4. Conclusions

An efficient analytical method was developed and validated for determination of pesticides in water samples using the technique DµSPE for sample preparation followed by LC-MS/MS determination. The method integrated an innovative extraction procedure with very sensible analysis producing good results in terms of selectivity, precision, recovery, and quantification limit of 0.01 µg L^−1^, compatible with the legislation limits for water monitoring. The results demonstrated that the developed method presents satisfactory analytical performance for 28 pesticide residues from different classes, meeting the main validation parameters recommended by SANTE [49]. The linearity assessment showed adequate behavior of the instrumental response in the studied concentration range, while the detection and quantification limits obtained proved to be compatible with the determination of residues in aqueous matrices. The accuracy and precision, evaluated at different spiking levels, confirmed the robustness of the method, demonstrating its applicability for environmental monitoring purposes. The use of extracted curves proved to be fundamental for the adequate quantification of the compounds, since it allowed the incorporation of sample preparation steps into the calibration process, more faithfully reflecting the analytical behavior of the analytes and contributing to the reliability of the results.

In the application to river water samples, the presence of pesticide residues was observed in a significant portion of the samples analyzed, with emphasis on the herbicide atrazine and the fungicide tebuconazole, evidencing the occurrence of these compounds in aquatic environments under the influence of agricultural activities. These results reinforce the importance of validated and sensitive methods for monitoring water quality. Overall, the proposed method proved suitable for the multiresidue determination of pesticides in aqueous matrices, with outstanding sensitivity and selectivity. Furthermore, the adoption of strategies aligned with the principles of Green Analytical Chemistry contributes to the reduction in solventreduction of solvent consumption and waste generation.

## Figures and Tables

**Table 1:** Results for the coefficient of determination (r^2^), accuracy (recovery, %), and precision (relative standard deviation, RSD %) of the method at different spike levels.

Compounds	r^2^	Recovery % (Repeatability, RSD %)			Recovery % (Intermediate Prec., RSD %)		
		Spike Levels (μg L^−1^)			Spike Levels (μg L^−1^)		
		0.01	0.02	0.1	0.01	0.02	0.1
Atrazine	0.9966	81.0 (12.9)	78.8 (11.1)	77.2 (16.7)	88.3 (9.0)	90.2 (12.3)	72.5 (6.0)
Bromuconazole	0.9972	96.5 (10.2)	98.6 (11.6)	93.9 (9.8)	96.7 (13.6)	98.3 (8.9)	85.3 (9.7)
Carbaryl	0.9994	82.9 (8.1)	75.9 (13.4)	70.7 (13.6)	94.4 (7.4)	94.2 (17.9)	90.6 (14.3)
Clomazone	0.9992	91.8 (14.0)	105.4 (8.0)	96.5 (8.4)	94.0 (13.7)	92.5 (7.8)	86.8 (8.8)
Cyanazine	0.9998	76.3 (13.7)	72.6 (13.3)	73.4 (14.4)	61.2 (14.6)	76.8 (11.6)	84.0 (12.9)
Difenoconazole	0.9943	106.4 (8.8)	88.4 (13.9)	79.0 (15.9)	86.9 (4.2)	89.2 (12.2)	81.0 (14.9)
Epoxiconazole	0.9970	99.1 (10.4)	91.0 (13.2)	80.9 (15.0)	80.9 (9.3)	89.0 (11.8)	79.8 (13.5)
Fenarimol	0.9998	104.1 (8.3)	85.6 (12.2)	85.1 (13.0)	94.7 (10.0)	82.4 (9.4)	85.8 (11.4)
Fluquinconazole	0.9990	102.3 (18.1)	93.0 (14.6)	80.4 (17.4)	78.4 (21.5)	87.8 (14.9)	79.6 (16.8)
Imidacloprid	0.9928	81.3 (6.8)	86.7 (8.0)	82.1 (7.1)	81.3 (6.8)	92.2 (4.5)	94.2 (6.7)
Iprovalicarb	0.9963	98.5 (8.9)	83.6 (10.8)	76.2 (15.4)	84.3 (6.7)	88.8 (7.7)	79.9 (14.1)
Linuron	0.9919	87.7 (7.8)	76.2 (11.1)	71.2 (11.3)	74.7 (5.0)	76.9 (8.0)	70.1 (8.7)
Metalaxyl	0.9981	89.0 (9.0)	88.0 (4.8)	77.6 (14.3)	87.0 (14.4)	82.5 (4.0)	81.1 (13.6)
Methiocarb-sulfone	0.9993	88.9 (13.4)	90.0 (7.5)	86.9 (10.2)	94.5 (13.9)	90.2 (4.6)	86.8 (10.2)
Metsulfuron-methyl	0.9976	51.0 (12.3)	43.1 (13.5)	57.0 (12.5)	23.8 (15.5)	28.9 (19.5)	49.1 (2.4)
Monolinuron	0.9985	99.9 (10.3)	83.6 (17.5)	77.6 (15.5)	72.1 (9.5)	79.4 (18.5)	79.9 (13.8)
Myclobutanil	0.9951	88.9 (12.0)	85.7 (13.2)	78.6 (17.5)	75.8 (8.9)	88.4 (10.3)	77.3 (18.2)
Paraoxon-ethyl	0.9981	100.5 (15.1)	85.5 (14.8)	78.7 (16.1)	73.1 (17.7)	81.8 (16.6)	83.2 (16.1)
Prochloraz	0.9989	92.3 (12.3)	83.2 (14.4)	79.5 (14.2)	73.0 (13.4)	85.2 (12.0)	82.3 (12.2)
Propiconazole	0.9979	99.3 (7.3)	115.0 (15.1)	118.3 (11.4)	112.4 (10.4)	109.8 (5.7)	111.7 (15.5)
Propyzamide	0.9990	91.2 (10.7)	80.1 (15.4)	74.5 (17.0)	87.9 (11.1)	74.4 (6.6)	82.5 (17.5)
Simazine	0.9941	72.6 (8.5)	72.3 (9.4)	70.9 (15.9)	87.3 (6.6)	72.7 (10.1)	76.2 (6.6)
Tebuconazole	0.9983	95.3 (13.9)	86.3 (10.6)	77.5 (16.2)	84.0 (11.8)	95.1 (7.5)	79.8 (14.9)
Tetraconazole	0.9987	105.0 (10.4)	85.4 (8.7)	77.7 (11.5)	78.4 (9.3)	84.7 (4.3)	73.9 (8.1)
Thiamethoxam	0.9997	73.9 (16.4)	71.8 (7.9)	71.8 (10.6)	76.1 (13.8)	84.3 (5.8)	84.6 (11.5)
Triadimefon	0.9939	85.4 (18.0)	80.8 (15.8)	77.1 (14.3)	74.4 (17.6)	86.2 (14.7)	83.8 (11.9)
Triadimenol	0.9972	90.3 (16.7)	91.2 (9.2)	80.8 (13.9)	74.6 (16.1)	82.7 (11.4)	76.9 (16.1)
Trifloxystrobin	0.9981	105.3 (9.9)	89.2 (10.2)	90.1 (10.5)	81.2 (6.7)	85.4 (7.3)	83.4 (12.1)
Triflumizole	0.9947	83.2 (10.6)	75.6 (13.4)	74.2 (13.9)	73.4 (9.5)	77.6 (11.8)	77.5 (16.1)

Prec.: precision.

**Table 2:** Concentrations of pesticides determined in river water samples collected in the state of Rio Grande do Sul, Brazil.

	Concentration in µg L^−1^																			
Compounds	S01	S02	S03	S04	S05	S06	S07	S08	S09	S10	S11	S12	S13	S14	S15	S16	S17	S18	S19	S20
Atrazine	<LOQ	n.d.	n.d.	n.d.	<LOQ	0.053	1.42	n.d.	n.d.	<LOQ	n.d.	n.d.	n.d.	<LOQ	<LOQ	<LOQ	0.625	0.130	0.052	1.793
Carbaryl	n.d.	n.d.	<LOQ	n.d.	<LOQ	<LOQ	n.d.	n.d.	<LOQ	<LOQ	<LOQ	n.d.	n.d.	<LOQ	<LOQ	<LOQ	n.d.	<LOQ	n.d.	n.d.
Clomazone	<LOQ	<LOQ	<LOQ	<LOQ	<LOQ	<LOQ	<LOQ	<LOQ	<LOQ	<LOQ	<LOQ	<LOQ	n.d.	0.103	<LOQ	n.d.	n.d.	<LOQ	<LOQ	0.071
Difenoconazole	n.d.	n.d.	n.d.	n.d.	n.d.	n.d.	n.d.	n.d.	n.d.	<LOQ	n.d.	n.d.	n.d.	<LOQ	n.d.	n.d.	n.d.	<LOQ	n.d.	n.d.
Fenarimol	<LOQ	n.d.	n.d.	n.d.	n.d.	n.d.	n.d.	n.d.	n.d.	<LOQ	n.d.	n.d.	n.d.	<LOQ	n.d.	n.d.	n.d.	<LOQ	n.d.	n.d.
Fluquinconazole	n.d.	n.d.	n.d.	n.d.	n.d.	n.d.	n.d.	n.d.	n.d.	<LOQ	n.d.	n.d.	n.d.	<LOQ	n.d.	n.d.	n.d.	n.d.	n.d.	n.d.
Imidaclorprid	<LOQ	n.d.	<LOQ	<LOQ	n.d.	n.d.	<LOQ	n.d.	n.d.	n.d.	n.d.	n.d.	n.d.	<LOQ	n.d.	n.d.	n.d.	n.d.	<LOQ	0.032
Iprovalicarb	n.d.	n.d.	n.d.	n.d.	n.d.	n.d.	n.d.	n.d.	n.d.	<LOQ	n.d.	n.d.	n.d.	n.d.	n.d.	n.d.	n.d.	n.d.	n.d.	n.d.
Linuron	n.d.	n.d.	n.d.	n.d.	n.d.	n.d.	n.d.	n.d.	n.d.	<LOQ	n.d.	n.d.	n.d.	<LOQ	n.d.	n.d.	n.d.	n.d.	n.d.	n.d.
Metalaxyl	n.d.	n.d.	n.d.	n.d.	n.d.	<LOQ	<LOQ	n.d.	n.d.	n.d.	n.d.	n.d.	n.d.	n.d.	n.d.	n.d.	n.d.	<LOQ	<LOQ	n.d.
Monolinuron	n.d.	n.d.	n.d.	n.d.	n.d.	n.d.	n.d.	n.d.	n.d.	n.d.	<LOQ	n.d.	n.d.	n.d.	n.d.	n.d.	n.d.	<LOQ	n.d.	n.d.
Myclobutanil	<LOQ	<LOQ	<LOQ	n.d.	n.d.	n.d.	n.d.	n.d.	n.d.	<LOQ	n.d.	n.d.	n.d.	0.053	n.d.	n.d.	n.d.	n.d.	n.d.	n.d.
Paraoxon-ethyl	n.d.	n.d.	n.d.	n.d.	n.d.	n.d.	n.d.	n.d.	n.d.	<LOQ	n.d.	n.d.	n.d.	n.d.	n.d.	n.d.	n.d.	<LOQ	n.d.	n.d.
Prochloraz	n.d.	n.d.	n.d.	n.d.	n.d.	n.d.	n.d.	n.d.	n.d.	<LOQ	n.d.	n.d.	n.d.	n.d.	n.d.	n.d.	n.d.	n.d.	n.d.	n.d.
Propyzamide	n.d.	n.d.	n.d.	n.d.	n.d.	n.d.	n.d.	n.d.	n.d.	<LOQ	n.d.	n.d.	n.d.	n.d.	n.d.	n.d.	n.d.	n.d.	n.d.	n.d.
Propiconazole	<LOQ	n.d.	<LOQ	<LOQ	n.d.	n.d.	<LOQ	n.d.	n.d.	<LOQ	n.d.	n.d.	n.d.	<LOQ	n.d.	<LOQ	n.d.	<LOQ	<LOQ	<LOQ
Simazine	n.d.	n.d.	n.d.	n.d.	n.d.	<LOQ	0.250	n.d.	n.d.	<LOQ	n.d.	n.d.	n.d.	n.d.	n.d.	<LOQ	n.d.	<LOQ	<LOQ	0.365
Tebuconazole	0.074	n.d.	<LOQ	<LOQ	<LOQ	<LOQ	<LOQ	n.d.	n.d.	<LOQ	n.d.	n.d.	n.d.	<LOQ	<LOQ	n.d.	0.066	<LOQ	0.26	0.034
Tetraconazole	n.d.	n.d.	n.d.	n.d.	n.d.	n.d.	<LOQ	n.d.	n.d.	<LOQ	n.d.	n.d.	n.d.	<LOQ	n.d.	n.d.	n.d.	<LOQ	n.d.	<LOQ
Thiamethoxam	<LOQ	n.d.	n.d.	n.d.	n.d.	0.063	0.041	n.d.	n.d.	n.d.	n.d.	n.d.	n.d.	<LOQ	<LOQ	<LOQ	<LOQ	n.d.	0.181	<LOQ
Trifloxystrobin	n.d.	n.d.	n.d.	n.d.	<LOQ	n.d.	n.d.	n.d.	n.d.	0.032	n.d.	n.d.	n.d.	0.037	n.d.	n.d.	n.d.	<LOQ	n.d.	n.d.
Triflumizole	n.d.	n.d.	n.d.	n.d.	n.d.	n.d.	n.d.	n.d.	n.d.	<LOQ	n.d.	n.d.	n.d.	n.d.	n.d.	n.d.	n.d.	n.d.	n.d.	n.d.

n.d.: not detected; LOQ: limit of quantification.

## Data Availability

Data are contained within the article.

## Supplementary Materials

The following supporting information can be downloaded at: https://www.mdpi.com/article/10.3390/molecules31111826/s1, Figure S1: Total Ion Chromatogram (TIC) obtained by LC-MS/MS referring to the extracted curve point at the 0.1 µg L^−1^ level; Figure S2: LC-MS/MS TIC for atrazine in sample S20 (top), at the LOQ level (center) and blank sample (bottom); Table S1: Acquisition parameters for LC-MS/MS, including retention time (t_R_), acquisition window, transitions of quantification and confirmation, dwell time, and instrumental parameters DP, EP, CE and CXP; Table S2: Recovery results of the different sorbents evaluated in the dispersive extraction step; Table S3: Recovery results of the sorbent Oasis HLB using different elution solvents.
