# Supplementary material for: Development of a Dispersive µSPE Method for the Determination of Pesticide Residues in Water Samples by LC-MS/MS
Source: Molecules. 2026 May 26;31(11):1826. doi: 10.3390/molecules31111826 (PMC13257659; doi:10.3390/molecules31111826)
Supplement: Supplementary file 1 [file molecules-31-01826-s001.zip › molecules-4254905-supplementary.pdf]

Supplementary Materials for

Development of a Dispersive µSPE Method for the Determination of Pesticide Residues in Water Samples by LC-MS/MS

Gabrielle D. Pereira [1], Igor F. de Souza [1], Luana Floriano [1], Osmar D. Prestes [1], Renato Zanella [1,*]

[1] Chemistry Department, Federal University of Santa Maria (UFSM), Santa Maria 97105-900, RS, Brazil
* Correspondence: renato.zanella@ufsm.br

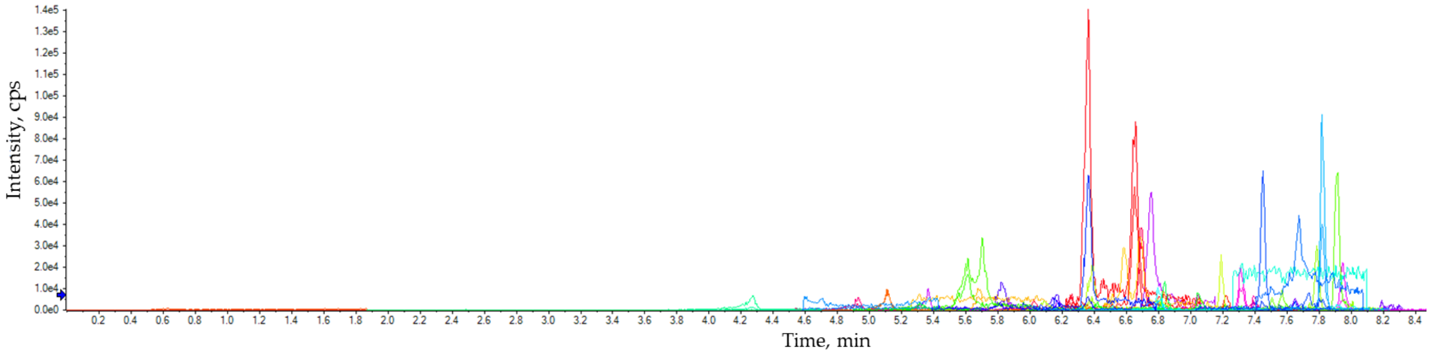

Supplementary Figure S1. Total Ion Chromatogram (TIC) obtained by LC-MS/MS referring to the extracted curve point at the 0.1 µg L^{-1} level.

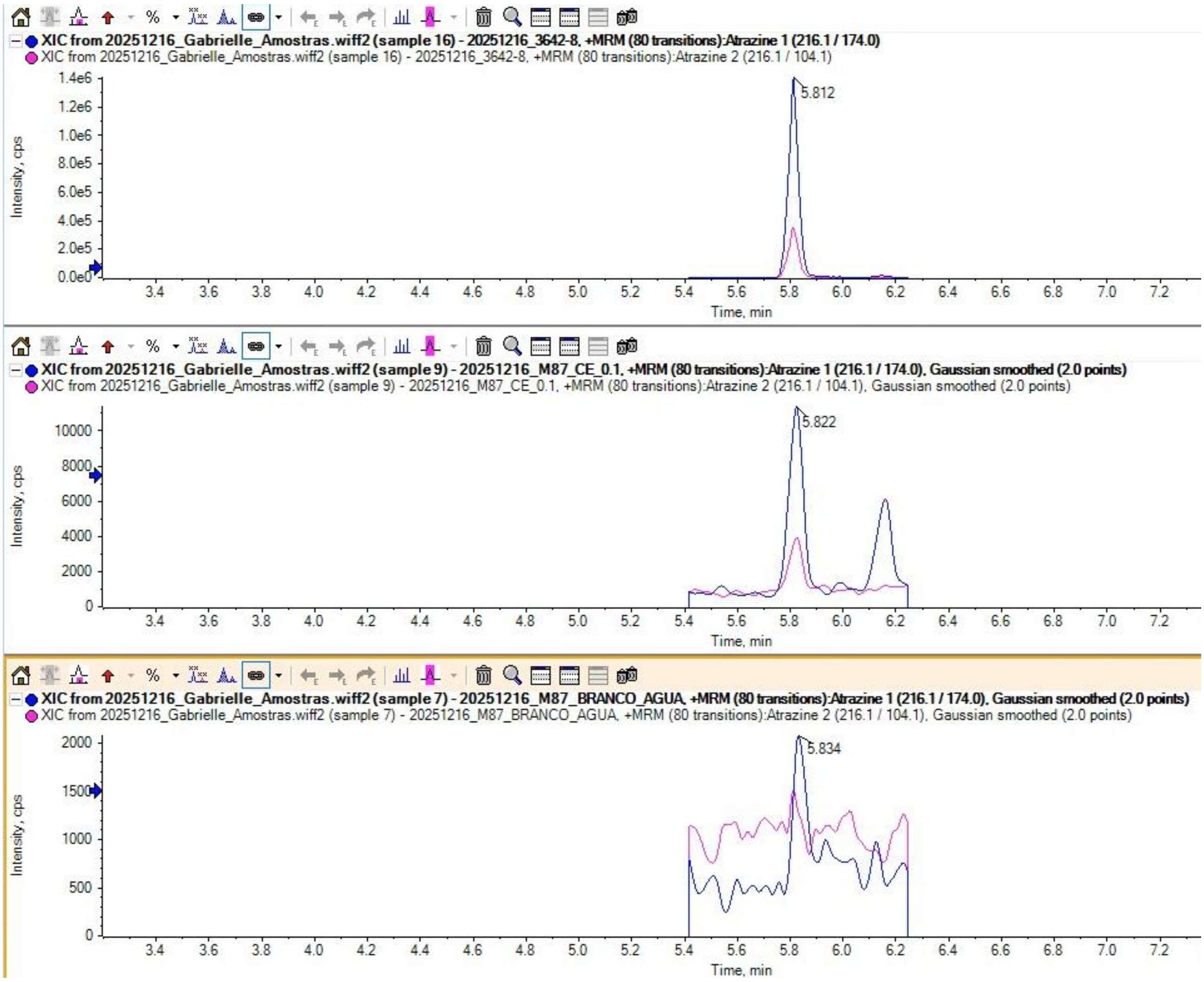

Supplementary Figure S2. LC-MS/MS TIC chromatogram for atrazine in sample S20 (top), at the LOQ level (centre) and blank sample (bottom).

Supplementary Table S1. Acquisition parameters for LC-MS/MS, including retention time (t_R), acquisition window, transitions of quantification and identification, dwell time, and instrumental parameters DP, EP, CE and CXP.

Compounds	t_R (min)	Acquisition window (s)	Transition (Da)		Dwell time (ms)	DP (V)	EP (V)	CE (eV)	CXP (V)
			Quantification	Confirmation					
Atrazine-d5	5.80	25	221.6	179.3	22.1	86	10	23	4
Atrazine-d5	5.81	25	221.6	101.4	22.0	86	10	31	4
Reserpine	7.20	25	609.3	195.1	10.7	100	10	38	11
Reserpine	7.20	25	609.3	397.1	10.7	100	10	47	11
Atrazine	5.83	25	216.1	174	21.9	86	10	23	4
Atrazine	5.83	25	216.1	104.1	21.9	86	10	39	4
Bromuconazole	7.65	40	378.1	161	22.9	47	10	39	10
Bromuconazole	7.65	40	378	159	22.9	91	10	35	10
Carbaryl	5.87	25	202.1	144.9	21.7	51	10	15	4
Carbaryl	5.88	25	202.1	127.1	21.7	71	10	39	4
Clomazone	6.73	25	240.1	125	10.8	66	10	27	4
Clomazone	6.73	25	240	89	10.8	66	10	65	4
Cyanazine	5.35	25	241.1	214.1	26.2	79	10	23	4
Cyanazine	5.36	25	241.2	104.1	26.1	81	10	39	4
Difenoconazole	7.94	25	406.1	251.1	38.9	81	10	37	4
Difenoconazole	7.94	25	406.1	337	38.9	81	10	26	4
Epoxiconazole	7.32	25	330.1	121	11.3	79	10	27	4
Epoxiconazole	7.32	25	330.1	101.1	11.3	79	10	63	4
Fenarimol	7.05	25	330.9	268	10.2	111	10	33	22
Fenarimol	7.04	25	330.9	189.1	10.2	111	10	65	18
Fluquinconazole	7.39	25	376	307.1	11.8	94	10	33	4
Fluquinconazole	7.39	25	376	349	11.8	91	10	25	4
Imidacloprid	4.94	25	256.1	209	43.7	89	10	23	4
Imidacloprid	4.93	25	256.1	175	44.4	89	10	25	4
Iprovalicarb	6.68	25	321.2	203.2	11.3	61	10	12	4
Iprovalicarb	6.70	25	321.2	119	11.1	89	10	23	4
Linuron	6.26	25	249	182.1	16.4	89	10	21	4
Linuron	6.26	25	249	159.9	16.4	89	10	23	4
Metalaxyl	6.64	25	280.1	220	11.7	71	10	19	4
Metalaxyl	6.65	25	280	248.1	11.6	66	10	15	16

Compounds	t_R (min)	Acquisition window (s)	Transition (Da)		Dwell time (ms)	DP (V)	EP (V)	CE (eV)	CXP (V)
			Quantification	Confirmation					
Metiocarb-sulfone	5.11	25	258.1	122.1	35.5	68	10	25	4
Metiocarb-sulfone	5.11	25	258.1	201.2	35.5	68	10	13	4
Metsulfuron-methyl	6.49	25	382	167	12.8	76	10	21	4
Metsulfuron-methyl	6.49	25	382.1	198.9	12.8	74	10	27	4
Monolinuron	5.68	25	215.1	125.9	23.1	79	10	25	4
Monolinuron	5.68	25	215.1	99	23.1	79	10	43	4
Myclobutanil	6.83	25	289.1	70.1	10.1	69	10	33	4
Myclobutanil	6.83	25	289.1	125.2	10.1	69	10	39	4
Paraoxon-ethyl	6.40	25	276	220	13.6	71	10	20	4
Paraoxon-ethyl	6.39	25	276	174	13.7	71	10	32	4
Prochloraz	7.78	25	376	308.1	20.3	64	10	17	4
Prochloraz	7.78	25	376	69.9	20.3	64	10	43	4
Propiconazole	7.56	25	342.1	159	14.3	86	10	43	4
Propiconazole	7.56	25	342.1	69.1	14.3	86	10	31	4
Propyzamide	6.42	25	256	190	13.4	81	10	19	4
Propyzamide	6.41	25	256	173.1	13.4	81	10	31	4
Simazine	5.32	25	202.1	132.1	26.8	86	10	25	4
Simazine	5.32	25	202.1	124.3	26.8	86	10	25	4
Tebuconazole	7.06	25	308.1	70.1	10.2	61	10	39	4
Tebuconazole	7.06	25	308.1	125	10.2	61	10	51	4
Tetraconazole	6.84	25	372	159	10.1	81	10	39	4
Tetraconazole	6.84	25	372	70	10.1	76	10	47	4
Thiamethoxam	4.28	25	292	211	83.1	74	10	17	4
Thiamethoxam	4.28	25	292	181	83.1	96	10	31	4
Triadimefon	6.81	25	294	197.2	10.2	76	10	21	4
Triadimefon	6.81	25	294	225	10.2	76	10	19	4
Triadimenol	6.46	25	296.1	70.1	13.0	56	10	19	4
Triadimenol	6.46	25	296	227	13.0	56	10	15	4
Trifloxystrobin	7.82	25	409.1	186.1	22.5	59	10	23	4
Trifloxystrobin	7.82	25	409	206	22.5	46	10	21	4
Triflumizole	7.45	25	346.1	278.1	12.5	16	10	17	12
Triflumizole	7.45	25	346.1	73	12.5	16	10	27	12

t_R: retention time; DP: declustering potential; EP: entrance potential; CE: collision energy, and CXP: collision cell exit potential.

Supplementary Table S2. Recovery results of the different sorbents evaluated in the dispersive extraction step.

**Compounds**	Recovery (%)				
	C18	Strata-X	Strata PRO	Bond Elut Plexa	Oasis HLB
Atrazine	7	103	104	76	99
Bromuconazole	74	58	55	48	75
Carbaryl	9	112	59	97	84
Clomazone	13	96	98	75	104
Cyanazine	4	91	92	67	112
Difenoconazole	30	115	101	88	115
Epoxiconazole	15	108	92	76	129
Fenarimol	12	95	82	77	112
Fluquinconazole	12	94	91	72	124
Imidacloprid	3	66	82	63	73
Iprovalicarb	7	104	95	63	122
Linuron	15	102	104	88	117
Metalaxyl	5	108	90	65	115
Methiocarb-sulfone	5	100	123	78	91
Metsulfuron-methyl	5	42	55	18	65
Monolinuron	7	94	116	82	111
Myclobutanil	10	107	93	72	128
Paraoxon-ethyl	6	107	102	83	113
Prochloraz	20	116	108	93	124
Propiconazole	14	128	107	87	111
Propyzamide	9	103	107	85	119
Simazine	4	92	107	70	105
Tebuconazole	12	102	92	76	121
Tetraconazole	16	122	115	82	118
Thiamethoxam	3	41	78	50	83
Triadimefon	8	108	94	77	110
Triadimenol	31	58	34	88	76
Trifloxystrobin	21	121	106	75	122
Triflumizole	25	124	122	79	115
**Recovered Compounds**	**1**	**25**	**25**	**26**	**29**

Supplementary Table S3. Recovery results of the sorbent Oasis HLB using diferente elution solventes.

**Compounds**	Recovery (%)					
	ACN	ACN 1% AA	MeOH	MeOH 1% AA	ACN:MeOH	ACN:MeOH 1% AA
Atrazine	72	75	71	73	74	78
Bromuconazole	69	102	105	101	76	86
Carbaryl	62	65	69	65	71	75
Clomazone	74	72	64	68	77	87
Cyanazine	75	77	75	75	72	78
Difenoconazole	98	83	62	64	79	82
Epoxiconazole	108	92	86	81	69	83
Fenarimol	71	70	79	76	85	84
Fluquinconazole	60	58	64	63	91	80
Imidacloprid	71	72	90	86	65	87
Iprovalicarb	57	62	71	67	39	77
Linuron	65	69	51	56	69	70
Metalaxyl	82	90	95	94	34	76
Methiocarb-sulfone	57	55	57	67	96	88
Metsulfuron-methyl	35	46	55	49	35	48
Monolinuron	96	58	71	77	86	81
Myclobutanil	91	101	104	79	72	78
Paraoxon-ethyl	78	57	67	74	93	82
Prochloraz	70	74	68	68	74	81
Propiconazole	66	72	84	103	91	110
Propyzamide	78	58	75	65	69	81
Simazine	62	83	77	68	74	75
Tebuconazole	65	77	72	79	80	80
Tetraconazole	76	64	81	82	78	75
Thiamethoxam	65	68	72	94	73	79
Triadimefon	72	73	74	74	78	84
Triadimenol	65	62	62	62	73	78
Trifloxystrobin	62	65	65	65	61	88
Triflumizole	79	64	68	65	63	77
**Recovered Compounds**	**26**	**23**	**26**	**27**	**26**	**28**
